# Quality of Life of Patients on Home Parenteral Nutrition Using Portable Infusion Pumps: A Cross-Sectional Study

**DOI:** 10.3390/nu18142236

**Published:** 2026-07-09

**Authors:** Mirosław Perliński, Wanda Danilewicz, Michał Frączek, Jacek Sobocki

**Affiliations:** 1 Faculty of Pharmacy, University of Vizja, 01-043 Warsaw, Poland; 2 Department of General Surgery, and Clinical Nutrition, The Centre of Postgraduate Medical Education, Czerniakowska 231, 00-416 Warsaw, Poland; 3 Center of Radiological Diagnostics, Clinical Department of Radiology, Radiotherapy and Nuclear Medicine, National Medical Institute of the Ministry of the Interior and Administration, 02-507 Warszawa, Poland

**Keywords:** home parenteral nutrition, portable infusion pumps, quality of life, nutrition

## Abstract

Background and Aims: Home parenteral nutrition (HPN) is a life-sustaining therapy for patients with chronic intestinal failure who cannot meet their nutritional needs enterally. Portable infusion pumps allow HPN to be administered safely at home, improving mobility and independence. However, their effect on quality of life (QoL) remains insufficiently explored. This study evaluated QoL in adults receiving HPN with portable infusion pumps at home. Methods: A cross-sectional survey was conducted in 102 adults receiving HPN with portable infusion pumps. Data were collected between September 2020 and December 2025 through outpatient clinics and a patient support group. Instruments included the WHOQOL-BREF and an author-designed questionnaire. Descriptive statistics, Mann-Whitney U, Kruskal-Wallis tests, and Pearson correlations were applied. Results: Overall QoL was moderate. WHOQOL-BREF scores were higher in the physical and psychological domains and lower in the social relationships and environmental domains. Most patients reported that portable pumps improved mobility, independence, and the ability to perform daily and social activities, including travel. Pump-related factors were positively associated with self-reported health status and overall QoL (*p* < 0.05). The main burdens were nocturnal infusions, sleep disturbances, and prolonged infusion duration. Metabolic complications and unplanned hospitalizations were infrequent and were not associated with pump usability. Conclusions: Portable infusion pumps may improve QoL in most patients receiving HPN by enhancing daily functioning and independence. Further optimization of pump-related factors may improve patient well-being, although some patients may not benefit from pump use. Clinical Relevance Statement: In adults receiving home parenteral nutrition, portable infusion pumps may improve daily functioning, independence, mobility, and participation in social and work-related activities. However, overall quality of life remains affected by treatment burden, particularly nocturnal infusions and prolonged infusion duration. These findings suggest that, in addition to selecting an appropriate infusion device, optimizing pump-related treatment factors may help improve patient-centered outcomes in home parenteral nutrition.

## 1. Introduction

Home parenteral nutrition (HPN) is a life-sustaining therapy for patients with chronic intestinal failure who are unable to meet nutritional requirements via the gastrointestinal tract. By enabling long-term survival outside the hospital, HPN has fundamentally changed the prognosis of these individuals. Nevertheless, the therapy remains complex and highly technology-dependent, requiring central venous access, strict aseptic procedures, and prolonged infusions that may interfere with daily activities, sleep, professional functioning, and social participation. In addition to the practical burden of prolonged infusion time, nocturnal administration of parenteral nutrition may interfere with sleep quality and normal circadian rhythmicity. Unlike physiological oral food intake, which is usually limited to daytime periods and follows regular digestive and metabolic cycles, long overnight intravenous nutrient delivery may alter the temporal pattern of substrate availability. The extent to which prolonged nocturnal infusions affect sleep, circadian regulation, metabolic homeostasis, and overall well-being in patients receiving HPN remains insufficiently studied. Consequently, quality of life (QoL) has become a key outcome in the evaluation of HPN, complementing traditional clinical endpoints [1,2,3,4].

The technology used for parenteral nutrition delivery has evolved substantially over the past decades. Early home programs relied on gravity-based systems or large stationary infusion devices, whereas later systems increasingly used electrically powered pumps providing controlled flow through the infusion tubing. Although stationary pumps improved infusion accuracy, they introduced practical burdens: dependence on continuous electrical supply throughout a 10–12-h infusion, limited battery capacity, difficult maneuvering of heavy IV poles, and confinement to the home during infusion hours. These constraints were particularly problematic for patients wishing to remain active, mobile, or employed, and for those aiming to travel or socialize. Portable infusion pumps were developed to address these limitations by allowing ambulatory infusions, more flexible scheduling, and integration of treatment into everyday life, including education, professional work, home duties, and childcare, etc.

Despite the widespread adoption of portable pump systems, evidence regarding their impact on patient-reported QoL in real-world HPN settings is still limited. Previous research has mainly focused on patient preferences for specific pump modalities or on technical aspects of home infusion devices, often in contexts other than HPN. Studies more closely related to HPN suggest that lack of portability and nocturnal disturbances associated with stationary equipment may negatively affect QoL, while portable pumps may enhance independence and reduce several daily burdens. However, comprehensive evaluations linking portable pump use with multidimensional QoL measures and everyday functioning in adult HPN populations remain scarce [2,5,6,7].

Therefore, this cross-sectional study aimed to assess the QoL of adult patients receiving HPN with portable infusion pumps and to explore how pump use relates to selected aspects of daily life and therapy outcomes. QoL was evaluated using the WHOQOL-BREF questionnaire, a multidimensional tool widely applied in chronic disease populations, supplemented with an author-designed survey focused on pump-related experiences. We also examined patients’ perceptions of pump influence on social activity, travel opportunities, professional functioning, metabolic complications, and unplanned hospitalizations, as well as the frequency of service needs during home use. By combining standardized QoL assessment with practical, patient-centered evaluation of portable pump use, our study seeks to provide clinically relevant insight into the real-life benefits and challenges of modern HPN delivery systems. It is worth underlining that HPN in Poland is publicly funded, organized through specialized centers, with care provided in home-care conditions. Patients are usually qualified and followed by specialized nutrition centers, which provide medical supervision, training, monitoring, and coordination of home treatment. This model differs from systems in which care is primarily delivered through local shared-care arrangements or private home-infusion providers. National professional standards and education in clinical nutrition are supported by the Polish Societies for Clinical Nutrition, while selected academic centers also contribute to specialist training and advisory expertise. This organizational background is important when interpreting patient-reported outcomes, because quality of life in HPN may be influenced not only by the infusion device itself, but also by training, monitoring, logistics, delivery systems, and access to specialized support.

## 2. Materials and Methods

### 2.1. Study Design and Setting

This cross-sectional observational study was conducted among adult patients receiving home parenteral nutrition (HPN) with the use of portable infusion pumps. Data were collected between September 2020 and December 2025 in home-care conditions. Participants were recruited through outpatient clinics providing specialized nutritional support and through a national patient support group dedicated to individuals receiving HPN.

### 2.2. Participants

Eligible participants were adults (≥18 years) receiving long-term home parenteral nutrition administered using portable infusion pumps. Inclusion criteria comprised current HPN therapy in home conditions, use of a portable infusion pump, and voluntary participation in the study. Patients who did not provide informed consent or submitted incomplete questionnaires were excluded from the analysis.

A total of 102 patients met the inclusion criteria and were included in the final analysis. Participation was voluntary, and a convenience sampling method was applied.

### 2.3. Data Collection and Study Instruments

#### 2.3.1. Quality of Life Assessment

Quality of life was assessed using the Polish version of the WHOQOL-BREF questionnaire, a validated, generic instrument developed by the World Health Organization for the assessment of quality of life in adults. The Polish WHOQOL-BREF has previously undergone psychometric evaluation in a Polish population and has demonstrated acceptable validity and reliability. The instrument is also available through the WHOQOL resources of the World Health Organization. The WHOQOL-BREF consists of 26 items covering four domains: physical health, psychological health, social relationships, and environment, as well as two general questions assessing overall quality of life and perceived health status. Domain scores were calculated according to WHOQOL-BREF scoring instructions. Domain means were computed and transformed to standard domain scores (higher scores indicating better quality of life). The physical/somatic domain reflects pain, energy, sleep, mobility, daily activities, dependence on medical treatment, and work capacity. The psychological domain includes positive and negative feelings, self-esteem, body image, thinking and concentration, and personal beliefs. The social domain assesses personal relationships, social support, and sexual activity, whereas the environmental domain covers safety, home environment, financial resources, access to health care, information, leisure, physical environment, and transport.

#### 2.3.2. Author-Designed Questionnaire

An author-designed questionnaire was used to collect patient-reported data on sociodemographic characteristics and infusion pump-related experiences during home parenteral nutrition. The survey included items on sex, age category, education, and occupational status, as well as HPN duration. Pump-related questions assessed perceived impact on everyday functioning (e.g., facilitation of daily activities), social participation and travel, perceived health changes since pump-assisted HPN, and work-related functioning. Additional items addressed treatment- and device-related burden, including infusion duration (hours per bag), nocturnal tolerance (noise/light disturbance), concordance between programmed and actual infusion time (including the magnitude of discrepancy if present), battery operating time after charging, perceived difficulty in pump operation (with optional specification), occurrence of pump malfunctions, and service needs (routine service adequacy and additional repairs). The questionnaire also captured pump type/model and the use and comfort of a backpack system for carrying the infusion set. Responses were recorded using single-choice categorical options, yes/no items, and short open-ended numeric fields. The questionnaire was administered anonymously and completed voluntarily by the participants.

### 2.4. Ethical Considerations

The study was conducted in accordance with the principles of the Declaration of Helsinki. Due to the non-interventional, anonymous, and survey-based nature of the study, formal approval from a local ethics committee was not required under national regulations. All participants were informed about the purpose of the study and provided voluntary informed consent prior to participation.

### 2.5. Statistical Analysis

Statistical analysis was performed using IBM SPSS Statistics for Windows, version 23.0 (IBM Corp., Armonk, NY, USA). Descriptive statistics were used to summarize participants’ characteristics and questionnaire results. Group comparisons were performed using the Mann-Whitney U test (two groups) and the Kruskal-Wallis test (three or more groups). Pearson’s correlation coefficient (r) was used to assess linear associations between continuous variables only (e.g., infusion time and WHOQOL-BREF domain scores). A two-sided *p*-value of <0.05 was considered statistically significant.

## 3. Results

**Participant characteristics**

A total of **102 respondents** participated in the study. Women constituted **65.7%** of the sample (***n* = 67**), and men **34.3%** (***n* = 35**).

The largest age group was **31–50 years** (***n* = 50; 49%**), followed by **51–70 years** (***n* = 28**; 27.4%). Participants aged **20–30 years** and **>70 years** each accounted for **11.8%** (***n* = 12** per group).

Education levels were comparable for secondary (***n* = 38; 37.2%**) and higher education (***n* = 37; 36.2%**), while vocational education accounted for **26.6%** (***n* = 27**).

Regarding occupational status, **43%** were employed (***n* = 44**), **40%** were retired/on disability pension (***n* = 41**), and **17%** were not working (***n* = 17**).

**Home parenteral nutrition and infusion pump use**

Most respondents had used home parenteral nutrition (HPN) for **>1 year** (***n* = 64; 62.7%**), while **24%** (***n* = 25**) reported “several months” and **13.3%** (***n* = 13**) “one year”.

Nearly all participants reported that the infusion pump facilitated daily functioning (**96%**, *n* = 98) and enabled planning a one-week recreational trip (**96%**, *n* = 97).

Similarly, **95%** (***n* = 97**) stated that pump use supported social activity (e.g., social meetings)

Within the last year, **73.5%** (***n* = 75**) reported no hospitalizations, while **17.7%** (***n* = 18**) were hospitalized once, and **8.8%** (***n* = 9**) more than once. 

A perceived improvement in health status since initiating pump-assisted HPN was reported by **85.3%** (***n* = 87**).

In the study group, **51%** (***n* = 52**) declared being professionally active, and **84.3%** (***n* = 86**) indicated that pump-assisted HPN facilitated performance of work-related duties.

**Pump performance and practical aspects**

The mean infusion time for one nutrition bag was **15.8 h (SD = 2.23)**; the most frequently reported infusion time was **17 h** (**31.4%**, *n* = 32). See detailed information in Table 1.

Nighttime pump operation was described as bothersome by **42.1%** (***n* = 43**).

About half of the respondents reported that the infusion duration matched the programmed time (**51%**, *n* = 52). Among those reporting a mismatch, the most common discrepancy was **1 h** (**63.7%**, *n* = 65), with a mean discrepancy of 1 h (SD = 0.35). See detailed information in Table 2.

The mean battery operating time after charging was **18.1 h (SD = 9.63)**, most commonly **16–18 h (27.2%)** and **12–15 h (26.1%)**. See detailed information in Table 3.

Most participants reported no difficulties performing pump-related activities (**95.1%**, *n* = 97).

Pump malfunctions or disruptions at any time during use were reported by **77.4%** (***n* = 79**), and **40.2%** (***n* = 41**) indicated a need for additional servicing.

By self-report, **67.7%** used the **Ambix Activ** pump (***n* = 69**), **28.4%** used **BD** (***n* = 29**), and **3.9%** used another device (***n* = 4**).

Regarding the use of a backpack for the nutrition set, **24.5%** (***n* = 25**) used it all day, **23.5%** (***n* = 24**) only when leaving home, **24.5%** (***n* = 25**) several days per week, and **27.5%** (***n* = 28**) did not use it. Among backpack users, **61.8%** (***n* = 63**) considered it comfortable.

Five patients returned the devices and did not wish to continue using them because of the additional burden of pump operation and the responsibility associated with expensive equipment, without perceiving a clear personal benefit.

**Quality of life (WHOQOL-BREF)**

The mean overall quality of life (1–5 scale) was **3.37 (SD = 0.75)**, while the mean self-rated health was **2.95 (SD = 0.95)**. Domain scores were: **somatic 19.77 (SD = 3.93)**, **psychological 18.61 (SD = 3.11)**, **social 9.77 (SD = 2.22)**, and **environmental 26.35 (SD = 4.92)**. See detailed information in Table 4.

**Sex**

Women scored higher than men in the **somatic domain** (**20.48 ± 3.92 vs. 18.44 ± 3.63**; **U = 713; *p* = 0.042**). No statistically significant sex differences were observed in other domains, overall quality of life, or health perception. See detailed information in Table 5.

**Age**

Quality of life differed across age groups. Significant differences were found for the **somatic** (**H = 11.1; *p* = 0.011**), **psychological** (**H = 16.2; *p* = 0.001**), and **environmental** domains (**H = 10.3; *p* = 0.016**), as well as **overall quality of life** (**H = 13.2; *p* = 0.004**) and **self-rated health** (**H = 9.4; *p* = 0.024**). The **social** domain did not differ significantly by age (**H = 4.7; *p* = 0.193**). The quality of life of people aged 20–30 and 31–50 is significantly higher than that of those aged 51 and older. People aged 20–30 and 31–50 are also significantly more satisfied with their health than those aged 51–70. See detailed information in Table 6.

**Education**

Respondents with vocational education reported lower **psychological** and **environmental** domain scores and lower **overall quality of life** compared with those with higher education (psychological: **H = 10.3; *p* = 0.006**; environmental: **H = 7.3; *p* = 0.025**; overall QoL: **H = 8.0; *p* = 0.018**). Other outcomes did not differ significantly by education level. See detailed information in Table 7.

**Occupational status and activity**

No significant differences in WHOQOL-BREF outcomes were observed across occupational status categories (employed vs. unemployed vs. retired/on disability pension). When comparing professionally active vs. inactive respondents, there was a **non-significant trend** toward higher overall quality of life among active participants (**U = 837; *p* = 0.062**). See detailed information in Table 8.

**Duration of home parenteral nutrition**

The somatic domain differed by duration of HPN use (**H = 7.1; *p* = 0.028**), with lower somatic scores in participants using HPN for “several months” compared with those using it for ≥1 year. Other domains and global ratings were not statistically significant. See detailed information in Table 9.

**Nighttime pump burden**

Participants who found nighttime pump operation burdensome reported significantly lower scores in the **psychological** (**U = 670; *p* = 0.004**), **social** (**U = 625; *p* = 0.001**), and **environmental** domains (**U = 587; *p* < 0.001**). Differences in overall quality of life showed a trend (***p* = 0.073**), while the somatic domain and health perception were not significant. See detailed information in Table 10.

**Pump type and backpack use**

Quality of life did not differ significantly by pump type (Ambix Activ vs. BD) across any WHOQOL-BREF outcomes. Backpack use frequency was associated with overall quality of life (**H = 9.8; *p* = 0.020**), with the highest quality-of-life ratings among those using the backpack all day. Other domains were not statistically significant. See detailed information in Table 11.

**Correlations**

Longer infusion time was associated with lower **psychological** domain scores (**r = −0.433; *p* < 0.001**) and lower **environmental** domain scores (**r = −0.280; *p* = 0.007**), as well as worse **self-rated health** (**r = −0.275; *p* = 0.008**). Correlations with the somatic domain, social domain, and overall quality of life were not statistically significant. See detailed information in Table 12.

## 4. Discussion

This cross-sectional study suggests that the use of portable infusion pumps in home parenteral nutrition (HPN) may improve the practical experience of treatment in everyday life, although overall quality of life remains only moderate. Most respondents reported that pump-assisted HPN facilitated daily functioning, social participation, travel, and work-related activity, indicating that portability may help patients preserve autonomy and maintain important life roles. This is consistent with previous reports showing that independence, mobility, and the ability to integrate HPN into daily routines are highly valued by patients receiving long-term nutrition support [2,3,4,5,6,8].

At the same time, our WHOQOL-BREF findings indicate that improved mobility does not fully remove the broader burden of long-term HPN. Although physical and psychological domain scores were relatively higher, the social relationships domain remained less favorable. This finding supports earlier observations that HPN, even when delivered with more flexible technology, remains a demanding and highly medicalized therapy that may continue to affect interpersonal relationships, spontaneity, and the sense of normal daily living [3,4,5,6,9].

An important observation in the present study is that treatment burden appeared to be more strongly associated with quality of life than the pump model itself. We did not observe significant differences between pump types, whereas nighttime burden and longer infusion duration were associated with poorer patient-reported outcomes. Participants who perceived nocturnal pump use as bothersome had lower psychological, social, and environmental domain scores, and longer infusion duration was correlated with poorer psychological and environmental scores as well as worse self-rated health. These findings are clinically plausible and consistent with previous literature indicating that treatment intrusiveness, sleep disturbance, and infusion schedule intensity are important determinants of patient experience during HPN [3,6,7].

We also found that patients using the backpack throughout the day reported the highest overall quality-of-life ratings. Although this association should not be interpreted causally, it may suggest that better integration of infusion equipment into routine mobility supports adaptation to treatment. Alternatively, patients with better baseline functioning may be more likely to use ambulatory carrying systems more extensively. Nevertheless, this finding highlights the practical importance of user-friendly accessories and individualized education regarding pump use in daily life.

The subgroup analyses suggest that quality of life may also be influenced by patient-related factors beyond the technical aspects of infusion delivery. Younger respondents reported better outcomes in selected QoL domains and in self-rated health, whereas respondents with vocational education had less favorable psychological and environmental scores. These findings should be interpreted cautiously, but they may reflect differences in functional reserve, social support, health literacy, or adaptation strategies. In contrast, pump type and occupational status were not significantly associated with WHOQOL-BREF outcomes, further suggesting that the patient experience of HPN depends more on treatment burden and adaptation than on device category alone.

The present study has several strengths. It combines a validated multidimensional quality-of-life instrument with a patient-centered questionnaire focused on real-world pump-related experiences. This approach allowed assessment not only of general QoL, but also of practical issues such as travel, mobility, work, nocturnal burden, and device usability. However, several limitations should be acknowledged. First, the cross-sectional design precludes causal inference. Second, the study was based on self-reported data and a convenience sample, which may have introduced response and selection bias. Third, no comparison group using stationary pumps or gravity-based systems was included. Fourth, the reasons for selecting daytime versus nocturnal infusion schedules were not systematically collected. Therefore, we could not determine whether infusion timing was driven primarily by patient preference, mobility, work-related needs, sleep tolerance, nursing routines, clinical considerations, or attempts to improve daily functioning. Finally, WHOQOL-BREF is a generic rather than HPN-specific instrument and may not capture all dimensions of treatment burden relevant to chronic intestinal failure [8,10,11].

Overall, our findings suggest that portable infusion pumps can improve the functional experience of HPN, particularly by enhancing mobility, independence, and participation in daily activities. However, quality of life remains strongly influenced by modifiable aspects of treatment delivery, especially nocturnal burden and infusion duration. From a clinical perspective, optimization of pump-assisted HPN should therefore extend beyond technical reliability and include efforts to reduce nighttime inconvenience, shorten infusion time where feasible, and better tailor device use to individual patient needs and preferences. Future prospective studies should determine whether such targeted interventions translate into measurable improvements in patient-reported outcomes.

## 5. Conclusions

Portable infusion pumps appear to improve the day-to-day experience of home parenteral nutrition in most patients by supporting mobility, independence, and participation in usual activities. However, the overall quality of life of patients receiving HPN remains influenced by the burden of treatment itself, particularly nocturnal inconvenience and prolonged infusion duration. These findings suggest that the benefit of portable pumps is not determined solely by device type, but also by how well therapy can be integrated into the patient’s daily life. Further prospective studies are needed to identify which technical and organizational modifications can most effectively improve patient-reported outcomes.

## Figures and Tables

**Table 1:** Feeding time.

How Many Hours Do You Spend on Parenteral Feeding?	N	%
7–12	12	11.8
13–15	20	19.6
16	20	19.6
17	32	31.4
18	13	12.7
19–20	5	4.9

**Table 2:** Compliance of the infusion time with the programmed time.

What Was the Difference Between Programmed and Real Infusion Time? (Hours)	N	%
0–0.5	52	51
0.5–1	8	7.8
1–1.5	32	31.4
1.5–2	8	7.8
2 or more	2	2

**Table 3:** Infusion pump operating time.

How Many Hours Does the Infusion Pump Run After Charging the Battery?	N	%
2–9	11	10.8
12–15	26	25.5
16–18	29	28.4
19–22	23	22.5
23–26	9	8.8
40–80	4	4

**Table 4:** Descriptive statistics for the individual domains of the WHOQOL-BREF questionnaire.

WHOQOL-BREF- Domain	M	SD	Min	Max
Somatic	19.77	3.93	11	28
Psychological	18.61	3.11	10	29
Social	9.77	2.22	4	14
Environmental	26.35	4.92	16	40
QoL	3.37	0.75	2	5
Self-rated health	2.95	0.95	1	5

**Table 5:** Descriptive statistics for the individual WHOQOL-BREF questionnaire scales by sex.

Sex	Women (N = 67)		Men (N = 35)		*U*	*p*
	*M*	*SD*	*M*	*SD*		
Somatic	20.48	3.92	18.44	3.63	713.0	0.042
Psychological	18.85	3.45	18.16	2.33	808.0	0.209
Social	9.82	2.21	9.69	2.26	936.0	0.842
Environmental	26.57	5.42	25.94	3.83	933.0	0.824
QoL	3.42	0.77	3.28	0.73	880.5	0.480
Self-rated health	2.97	0.92	2.91	1.03	933.5	0.820

**Table 6:** Descriptive statistics for the individual WHOQOL-BREF questionnaire scales by age.

Age	20–30 y.o. (N = 12)		31–50 y.o. (N = 50)		51–70 y.o. (N = 28)		>70 y.o. (N = 12)		*H*	*p*
	*M*	*SD*	*M*	*SD*	*M*	*SD*	*M*	*SD*		
Somatic	21.00	4.24	20.84	3.46	18.31	3.79	17.73	4.22	11.1	0.011
Psychological	20.91	3.78	19.20	2.54	17.00	3.17	17.73	2.41	16.2	0.001
Social	9.82	1.99	10.23	2.32	9.35	2.08	8.91	2.17	4.7	0.193
Environmental	29.27	6.66	27.30	4.48	24.38	3.93	24.27	4.73	10.3	0.016
QoL	3.91	0.70	3.50	0.70	3.08	0.74	3.00	0.63	13.2	0.004
Self-rated health	3.36	1.12	3.14	0.88	2.50	0.91	2.82	0.87	9.4	0.024

**Table 7:** Descriptive statistics for the individual WHOQOL-BREF questionnaire scales by education.

Education	Vocational (N = 27)		Secondary (N = 38)		Tertiary (N = 37)		*H*	*p*
	*M*	*SD*	*M*	*SD*	*M*	*SD*		
Somatic	18.68	4.38	19.94	3.88	20.42	3.54	2.8	0.248
Psychological	17.04	3.36	19.06	2.62	19.33	3.05	10.3	0.006
Social	9.48	2.29	9.50	2.14	10.27	2.23	2.4	0.304
Environmental	24.16	4.16	26.76	4.84	27.58	5.11	7.3	0.025
QoL	3.04	0.79	3.33	0.73	3.64	0.65	8.0	0.018
Self-rated health	2.88	1.09	3.03	0.87	2.91	0.95	0.5	0.783

**Table 8:** Descriptive statistics for the individual WHOQOL-BREF questionnaire scales by professional activity.

Professional Activity	Yes (N = 44)		No (N = 17)		Retired (N = 41)		*H*	*p*
	*M*	*SD*	*M*	*SD*	*M*	*SD*		
Somatic	19.92	3.90	19.81	3.90	19.59	4.06	0.3	0.845
Psychological	18.90	2.51	17.94	4.85	18.59	2.77	1.1	0.590
Social	9.92	2.14	9.31	2.65	9.81	2.13	0.3	0.849
Environmental	26.77	4.69	25.25	4.63	26.38	5.31	1.1	0.586
QoL	3.46	0.68	3.19	0.83	3.35	0.79	1.7	0.420
Self-rated health	2.92	0.87	3.00	1.15	2.95	0.97	0.0	0.993

**Table 9:** Descriptive statistics for the individual WHOQOL-BREF questionnaire scales by duration of home parenteral nutrition use.

Time of Using HPN	Few Months (N = 25)		1 Year (N = 13)		Above 1 Year (N = 64)		*H*	*p*
	*M*	*SD*	*M*	*SD*	*M*	*SD*		
Somatic	17.83	3.52	21.17	3.79	20.20	3.89	7.1	0.028
Psychological	17.39	2.57	18.58	3.18	19.04	3.19	4.5	0.103
Social	8.91	1.76	10.00	2.73	10.07	2.24	5.1	0.079
Environmental	24.26	3.56	26.75	5.14	27.04	5.10	5.2	0.075
QoL	3.13	0.76	3.42	0.79	3.45	0.74	2.6	0.269
Self-rated health	2.57	0.95	3.00	1.04	3.07	0.91	4.5	0.107

**Table 10:** Descriptive statistics for the individual WHOQOL-BREF questionnaire scales by perceived burden of nighttime pump operation.

If Infusion Pump Runs at Night, Is It Bothersome?	Yes (N = 43)		No (N = 59)		*U*	*p*
	*M*	*SD*	*M*	*SD*		
Somatic	19.26	3.34	20.15	4.30	871.0	0.197
Psychological	17.51	2.45	19.42	3.31	670.0	0.004
Social	8.90	1.94	10.42	2.21	625.0	0.001
Environmental	24.18	4.11	27.94	4.88	587.0	0.000
QoL	3.18	0.79	3.51	0.70	824.5	0.073
Self-rated health	2.85	0.84	3.02	1.03	926.5	0.375

**Table 11:** Descriptive statistics for the individual WHOQOL-BREF questionnaire scales by backpack use.

How Often Do You Use Your Nutrition Kit Backpack?	All Day (N = 25)		Only When Leaving Home (N = 24)		Several Days per Week (N = 25)		I Don’t Use a Backpack (N = 28)		*H*	*p*
	*M*	*SD*	*M*	*SD*	*M*	*SD*	*M*	*SD*		
Somatic	20.71	2.91	19.11	3.83	20.14	4.38	19.36	4.37	3.2	0.362
Psychological	19.46	2.30	18.26	2.08	17.68	2.57	18.92	4.48	3.3	0.342
Social	9.42	2.08	9.53	2.25	10.18	2.24	9.96	2.41	2.0	0.572
Environmental	26.33	4.20	25.42	4.41	27.45	3.40	26.12	6.91	2.9	0.413
QoL	3.75	0.44	3.16	0.69	3.41	0.67	3.20	0.96	9.8	0.020
Self-rated health	3.21	0.83	2.53	0.77	3.05	0.95	3.00	1.08	6.1	0.106

**Table 12:** Pearson r correlations between individual domains and feeding bag transfusion time.

		Infusion Time
Somatic	*r*	−0.148
	*p*	0.163
Psychological	*r*	−0.433 **
	*p*	0.000
Social	*r*	−0.197
	*p*	0.061
Environmental	*r*	−0.280 **
	*p*	0.007
QoL	*r*	−0.144
	*p*	0.173
Self-rated health	*r*	−0.275 **
	*p*	0.008

** *p* < 0.01.

## Data Availability

The data presented in this study are available on reasonable request from the corresponding author. The data are not publicly available due to privacy and ethical restrictions, as they contain patient-reported information related to health status, home parenteral nutrition, and quality of life.
